# STAT1-deficient mice spontaneously develop estrogen receptor α-positive luminal mammary carcinomas

**DOI:** 10.1186/bcr3100

**Published:** 2012-01-20

**Authors:** Szeman Ruby Chan, William Vermi, Jingqin Luo, Laura Lucini, Charles Rickert, Amy M Fowler, Silvia Lonardi, Cora Arthur, Larry JT Young, David E Levy, Michael J Welch, Robert D Cardiff, Robert D Schreiber

**Affiliations:** 1 Department of Pathology and Immunology, Washington University School of Medicine, 425 S. Euclid Avenue, St. Louis, MO 63110, USA; 2 Department of Pathology, University of Brescia/Spedali Civilli di Brescia, Piazzale Spedali Civili 1, Brescia 25123, Italy; 3 Division of Biostatistics, Washington University School of Medicine, 660 S. Euclid Avenue, St. Louis, MO 63110, USA; 4 Division of Radiological Sciences, Edward Mallinckrodt Institute of Radiology, 660 S. Euclid Avenue, St. Louis, MO 63110, USA; 5 Center for Comparative Medicine, Department of Pathology and Laboratory Medicine, University of California Davis, County Road 98 and Hutchison Drive, Davis, CA 95616, USA; 6 Department of Pathology, New York University School of Medicine, 550 First Avenue, MSB 548, New York, NY 10016, USA

## Abstract

**Introduction:**

Although breast cancers expressing estrogen receptor-α (ERα) and progesterone receptors (PR) are the most common form of mammary malignancy in humans, it has been difficult to develop a suitable mouse model showing similar steroid hormone responsiveness. STAT transcription factors play critical roles in mammary gland tumorigenesis, but the precise role of STAT1 remains unclear. Herein, we show that a subset of human breast cancers display reduced STAT1 expression and that mice lacking STAT1 surprisingly develop ERα+/PR+ mammary tumors.

**Methods:**

We used a combination of approaches, including histological examination, gene targeted mice, gene expression analysis, tumor transplantaion, and immunophenotyping, to pursue this study.

**Results:**

Forty-five percent (37/83) of human ERα+ and 22% (17/78) of ERα- breast cancers display undetectable or low levels of STAT1 expression in neoplastic cells. In contrast, STAT1 expression is elevated in epithelial cells of normal breast tissues adjacent to the malignant lesions, suggesting that STAT1 is selectively downregulated in the tumor cells during tumor progression. Interestingly, the expression levels of STAT1 in the tumor-infiltrating stromal cells remain elevated, indicating that single-cell resolution analysis of STAT1 level in primary breast cancer biopsies is necessary for accurate assessment. Female mice lacking functional STAT1 spontaneously develop mammary adenocarcinomas that comprise > 90% ERα+/PR+ tumor cells, and depend on estrogen for tumor engraftment and progression. Phenotypic marker analyses demonstrate that STAT1^-/- ^mammary tumors arise from luminal epithelial cells, but not myoepithelial cells. In addition, the molecular signature of the STAT1^-/- ^mammary tumors overlaps closely to that of human luminal breast cancers. Finally, introduction of wildtype STAT1, but not a STAT1 mutant lacking the critical Tyr701 residue, into STAT1^-/- ^mammary tumor cells results in apoptosis, demonstrating that the tumor suppressor function of STAT1 is cell-autonomous and requires its transcriptional activity.

**Conclusions:**

Our findings demonstrate that STAT1 suppresses mammary tumor formation and its expression is frequently lost during breast cancer progression. Spontaneous mammary tumors that develop in STAT1^-/- ^mice closely recapitulate the progression, ovarian hormone responsiveness, and molecular characteristics of human luminal breast cancer, the most common subtype of human breast neoplasms, and thus represent a valuable platform for testing novel treatments and detection modalities.

## Introduction

Estrogen receptor-alpha-positive (ERα^+^) and progesterone receptor-positive (PR^+^) breast cancer account for approximately 60% to 70% of the breast cancer cases diagnosed in humans [1,2]. The majority of these tumors exhibit a molecular signature that is characteristic of the luminal subtype [3]. The standard of care for luminal breast cancer is either to inhibit ERα signaling using selective ER modulators or to deprive the tumors of estradiol (E2) by ovarian ablation or aromatase inhibition [4]. Despite the advances in the treatment of luminal breast cancers, progress has been hampered by a significant deficit in murine models that fully reproduce the hormonal responsiveness and dependency of human ERα^+^/PR^+ ^breast cancers [5-8] and that can be used to develop better methods to follow the disease after treatment.

STAT1 is a transcription factor that plays a critical role in interferon (IFN) signaling [9]. Cells lacking STAT1 respond aberrantly to IFNα/β and IFNγ, and STAT1^-/- ^mice display immune defects rendering them highly susceptible to infection [10,11] and tumor development [12,13]. The latter finding shows that STAT1 is important in manifesting the IFN-dependent, cell-extrinsic tumor suppressor actions of immunity (that is, the elimination phase of cancer immunoediting [14]). Other studies have also suggested that STAT1 can function as a cell-intrinsic tumor suppressor by maintaining basal expression levels of caspases [15], upregulating p27^Kip1 ^expression [16,17], or interacting with p53 or BRCA1 [18-20]. However, these latter studies were conducted mostly with cell lines *in vitro *and have not been validated by *in vivo *approaches. Most recently, *in vivo *studies indicated that STAT1 could suppress tumor development in the ErbB2/Neu-driven mammary tumor models [21,22], although its action in other types of mammary tumors remains undefined. Paradoxically, others have proposed that STAT1 can facilitate tumor outgrowth since elevated levels of STAT1 in melanoma cell lines result in their acquisition of resistance to radiation or chemotherapy [23,24]. This apparent paradox has also been observed in biopsies of human breast cancers [25,26]. However, it remains unclear whether the altered STAT1 levels were present in the breast cancer cells themselves or in stromal cells. Thus, the physiological role of STAT1 during cancer development remains poorly understood and may be context-dependent.

Here, we show that STAT1 expression is lost or significantly diminished in the neoplastic cells of a subset of human patients with ERα^+^/PR^+ ^breast cancer relative to normal breast epithelium, suggesting that downregulation of STAT1 is associated with tumor progression. To further investigate this observation, we followed female mice lacking STAT1 longitudinally and found that they spontaneously develop ERα^+^/PR^+^, hormone-responsive mammary gland cancers of the luminal subtype, thus closely recapitulating the characteristics of human ERα^+^/PR^+ ^luminal breast cancers.

## Materials and methods

### Immunohistochemistry on human breast cancer samples

**Figure 1:**
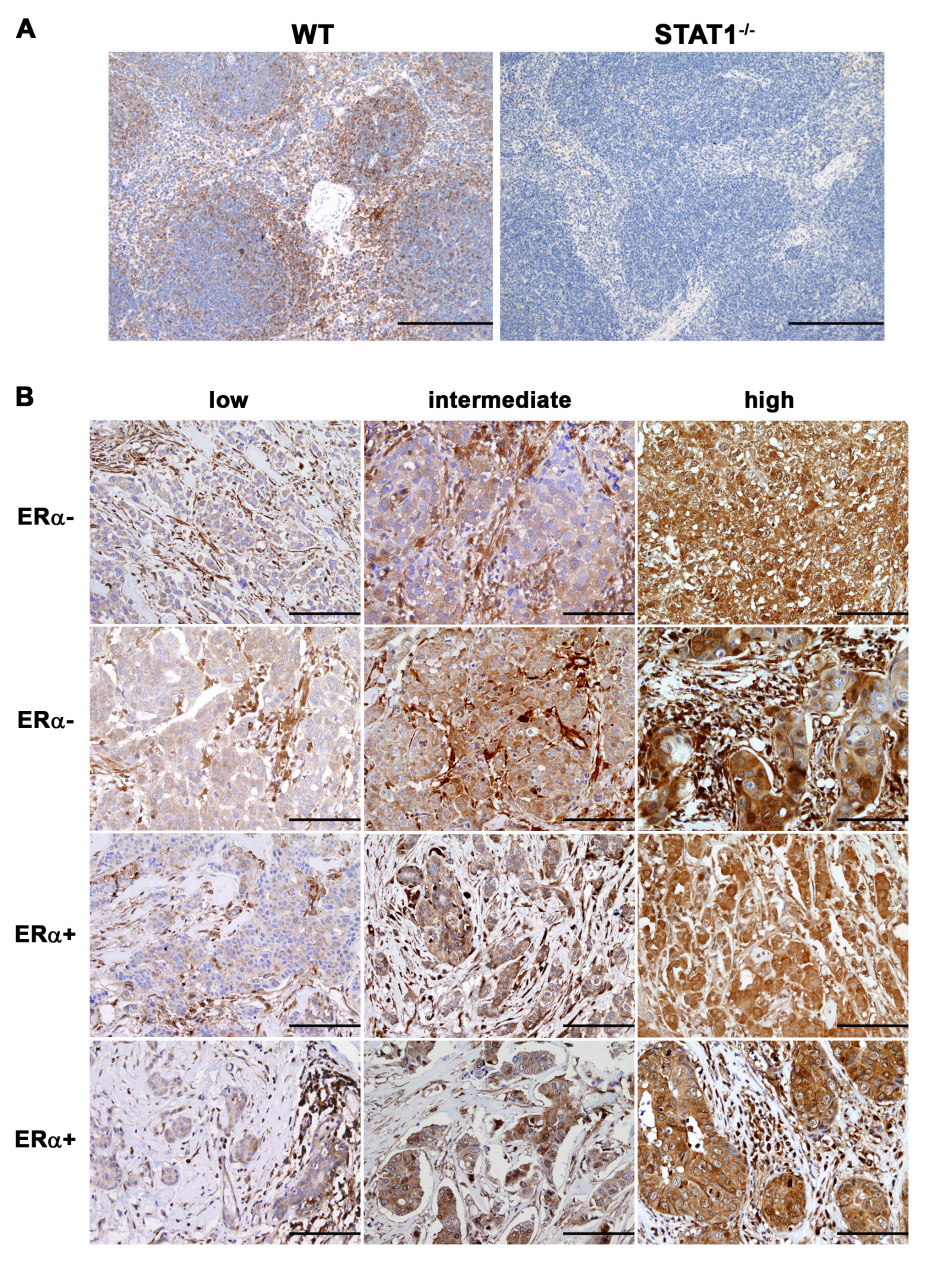
**Immunohistochemical analysis of STAT1 expression levels in human breast cancers**. **(A) **Immunohistochemical analysis on wild-type (WT) and STAT1^-/- ^spleens to confirm the specificity of the antibody against STAT1. Sections of WT or STAT1^-/- ^spleens were stained with a polyclonal rabbit antibody against an epitope in STAT1 which is homologous to both humans and mice. STAT1 reactivity was observed predominantly in the lymphoid cells of the white pulp in WT spleen. However, no appreciable signal was detected in the STAT1^-/- ^spleen. Original magnification, 100×. Scale bars = 200 μm. **(B) **The staining intensity of the STAT1^+ ^signal was evaluated by using a three-tiered scale (low, intermediate, and high). Low, intermediate, and high STAT1 correspond to the staining intensity scores of 1, 2, and 3, respectively, in Table 2. Six cases of estrogen receptor-alpha-negative (ERα^-^) and six cases of ERα^+ ^primary human breast tumors are shown here as representative images. Original magnification, 200×. Scale bars = 100 μm.

Formalin-fixed paraffin-embedded tissue blocks were retrieved from the archive of the Department of Pathology in Spedali Civili di Brescia, Brescia, Italy. This retrospective study was conducted in compliance with the Declaration of Helsinki and with policies approved by the Ethics Board of Spedali Civili di Brescia. For this large-scale retrospective and exclusively observational study on archival materials, patient consent was not needed, as established by Italian regulations (Delibera del garante n. 52 del 24/7/2008 and DL 193/2003). The cohort consisted of 161 primary breast carcinomas selected from a series of routinely examined cases collected between 2006 and 2008, adjacent normal breast tissues from 11 patients with cancer, and normal breast tissues from five healthy individuals (tissues kindly provided by Monica Guaragni, Clinica S. Anna, Brescia, Italy). Breast carcinoma samples were characterized on the basis of histology, ERα (clone SP1; Thermo Fisher Scientific, Waltham, MA, USA) and PR (clone PgR 636; Dako, Glostrup, Denmark) expression, and HER2 amplification by immunohistochemistry (Herceptest; Dako) and fluorescence *in situ *hybridization (PathVision HER-2 DNA probe kit; Abbott Laboratories, Abbott Park, IL, USA) (summarized in Table 1). STAT1 protein expression was evaluated on four-micron (4-μm) tissue sections by using a rabbit polyclonal antibody against STAT1 (sc-346, 1:400; Santa Cruz Biotechnology, Inc., Santa Cruz, CA, USA). Antigen retrieval was performed by microwaving in citrate buffer (pH 6.0). Positive signal was revealed by the Super Sensitive polymer-horseradish peroxidase immunohistochemistry detection system in accordance with the instructions of the manufacturer (BioGenex, San Ramon, CA, USA). Mouse spleens obtained from WT and STAT1^-/- ^mice were used as positive and negative controls, respectively, to confirm the specificity of the STAT1 antibody (Figure 1A). STAT1 expression of the human normal breast tissues and breast tumor samples was assessed according to the percentage of STAT1^+ ^tumor or stromal cells (percentage score: 1 = fewer than 5% of positive cells; 2 = 5% to 25% of positive cells; 3 = 25% to 75% of positive cells; and 4 = greater than 75% of positive cells) and to the intensity of the staining (intensity score: 1 = low; 2 = intermediate, and 3 = high) (Table 1 and Figure 1B), similar to the determination of ERα expression by using the Allred score [27]. The percentage score was then added to the intensity score to produce the final STAT1 score.

**Table 1:** Clinicopathological characteristics of the human patients with breast cancer in this study with STAT1 staining results

	Number (percentage)			
	All cases	ER^-^	ER^+^	
Characteristics	(*n *= 161)	(*n *= 78)	(*n *= 83)	*P *value
PR expression				
Negative	95 (59%)	78 (100%)	7 (8%)	2.91 × 10^-38a^
Positive	76 (47%)	0 (0%)	76 (92%)	
HER2 IHC or FISH				
Negative	129 (80%)	46 (59%)	83 (100%)	1.35 × 10^-12a^
Positive	32 (20%)	32 (41%)	0 (0%)	
Histology group^b^				
A	6 (4%)	2 (3%)	4 (5%)	
B	138 (85%)	71 (91%)	67 (81%)	0.2512^a^
C	16 (10%)	5 (6%)	11 (13%)	
D	1 (1%)	0 (0%)	1 (1%)	
Percentage of STAT1^+ ^neoplastic cells (percentage score)				
< 5%	17 (11%)	7 (9%)	10 (12%)	
5%-25%	20 (12%)	10 (13%)	10 (12%)	0.91^c^
26%-75%	65 (40%)	33 (42%)	32 (39%)	
> 75%	59 (37%)	28 (36%)	31 (37%)	
Percentage of STAT1^+ ^stromal cells (percentage score)				
< 5%	0 (0%)	0 (0%)	0 (0%)	
5%-25%	16 (10%)	2 (3%)	14 (17%)	0.006^a^
26%-75%	54 (34%)	26 (33%)	28 (34%)	
> 75%	91 (56%)	50 (64%)	41 (49%)	
STAT1 intensity in STAT1^+ ^neoplastic cells (intensity score)				
Low	54 (34%)	17 (22%)	37 (45%)	
Intermediate	60 (37%)	31 (40%)	29 (35%)	0.0042^c^
High	47 (29%)	30 (38%)	17 (20%)	
STAT1 intensity in STAT1^+ ^stromal cells (intensity score)				
Low	2 (1%)	0 (0%)	2 (2%)	
Intermediate	18 (11%)	3 (4%)	15 (18%)	0.0027^a^
High	141 (88%)	75 (96%)	66 (80%)	

^a^Fisher exact test. ^b^Histology group A: ductal carcinoma *in situ *with minor areas of invasive ductal carcinoma; group B: invasive ductal carcinoma; group C: lobular carcinomas; group D: mixed ductal and lobular carcinomas. ^c^Chi-squared test. ER, estrogen receptor; FISH, fluoresence *in situ *hybridization; HER2, human epidermal growth factor receptor 2; IHC, immunohistochemistry; PR, progesterone receptor.

### Mice

129S6/SvEvTac-*Stat1^tm1Rds ^*(referred to here as STAT1-deficient, or STAT1^-/-^), 129S6/SvEvTac-*Stat1^tm1Rds^*-*Rag2 ^tm1Fwa ^*(referred to here as STAT1^-/- ^× RAG2^-/-^), and B6.129S-*Stat1^tm1Dlv ^*(referred to here as STAT1-null, or S1N) mice were generated and previously characterized in our laboratories [10,11,13]. RAG2^-/- ^mice were generated by Frederick Alt [28]. Wild-type (WT) 129S6/SvEv and STAT1^-/- ^(129S6/SvEv) mice were purchased from Taconic Farms (Hudson, NY, USA), and STAT1-null mice (mixed C57BL/6-129/SvEv and pure C57BL/6) were maintained and housed in our laboratories. Mice were scored as tumor-bearing when they reached 10 by 10 mm in size. All animal experiments were carried out in accordance with the guidelines of the American Association for Laboratory Animal Science as an approved protocol by the Animal Studies Committees in both the Washington University School of Medicine and New York University. These experiments were performed in specific pathogen-free facilities that were accredited by the Association for Assessment and Accreditation of Laboratory Animal Care and that were located at the Washington University School of Medicine and New York University School of Medicine.

### Cell cultures

STAT1^-/- ^mammary tumor cell lines, SSM1, SSM2, and SSM3, were originated from three individual STAT1^-/- ^tumor-bearing mice and were created by mechanical disaggregation of tissue before an overnight incubation with collagenase type IA (200 U/mL; Sigma-Aldrich, St. Louis, MO, USA) and insulin (10 μg/mL; Sigma-Aldrich) in DMEM/F12/10% FBS/1% L-glutamine/1% penicillin-streptomycin. Clusters of epithelial cells were enriched by sedimentation through five washes of 40 mL of DMEM/F12/5% FBS each. Stromal fibroblasts were eliminated by differential trypsinization by using 0.05% trypsin once a week over the next 2 months. The absence of fibroblasts was determined by immunofluoresence by using an antibody specific for vimentin (sc-7557; Santa Cruz Biotechnology, Inc.). The SSM cell lines are maintained in DMEM/F12/10% FBS/1% L-glutamine/1% penicillin-streptomycin/50 μM 2-mercaptoethanol/0.3 μM hydrocortisone/5 μg/mL insulin/10 ng/mL transferrin. MCF7 and NMuMG were purchased from American Type Culture Collection (Manassas, VA, USA) and are cultured in DMEM/10% FBS/1% L-glutamine/1% penicillin-streptomycin. The MCF7 cell line is a human ERα^+ ^breast cancer cell line. NMuMG is an immortalized nontransformed murine mammary gland epithelial cell line.

## Immunohistochemistry and immunofluorescence analyses on murine tumor samples

Tumors were harvested and fixed in 10% neutral buffered formalin for 1 to 2 days. Paraffin blocks were made, and slides were stained with hematoxylin and eosin (H&E) for histological examination. To examine the presence of ERα and PR, slides were deparaffinized, serially rehydrated, and stained in accordance with the routine procedures in the Mutant Mouse Pathology Laboratory of the University of California at Davis. For the immunofluoresence assay determining the presence of fibroblasts, SSM tumor cells were plated on coverslips and allowed to attach overnight. Cells were then fixed and permeablized with ice-cold methanol and then acetone for 10 minutes each at -20°C. Coverslips were washed extensively with 1× phosphate-buffered saline (PBS) and blocked with 5.5% normal donkey serum and 2% bovine serum albumin in PBS. Cells were incubated with anti-cytokeratin (anti-CK) (wide spectrum) (Z0622, 1:200; Dako) or anti-vimentin (sc-7557, 1:50; Santa Cruz Biotechnology, Inc.) for 1 hour at room temperature. Donkey anti-rabbit conjugated with Cy2 and donkey anti-goat conjugated with Cy3 (1:200; Jackson ImmunoResearch Laboratories, Inc., West Grove, PA, USA) were used for the detection of CK and vimentin, respectively. To examine the expression of ERα, tumor cells were plated on coverslips, fixed in 4% paraformaldehyde, and permeablized in 0.5% Triton X-100. Monoclonal antibody against ERα (6F11, 1:40; Novocastra, now part of Leica, Wetzlar, Germany) and donkey anti-mouse conjugated with Cy3 were used. DAPI (4'-6-diamidino-2-phenylindole) was used to identify nuclei in all immunofluoresence assays (Molecular Probes, now part of Life Technologies, Grand Island, NY, USA).

### Southern blotting

Southern blot analysis was performed as described previously [29]. The probe was prepared by digesting a plasmid containing the MMTV long terminal repeat with BamHI (the MMTV-LTR plasmid was a generous gift from Elena Buetti of the Swiss Institute for Experimental Cancer Research in Switzerland) [30].

### MTT

MCF7, SSM1, SSM2, or SSM3 was plated in 96-well plates in phenol red-free media with 10% charcoal-treated FBS (HyClone, Logan, UT, USA) supplemented with hydrocortisone, insulin, and transferrin and with or without 10 nM 17-β-estradiol (Sigma-Aldrich). Cell proliferation was determined by using 3-(4,5-dimethyl-2-thiazlyl)-2,5-diphenyl-2H-tetrasolium bromide (MTT) assays in accordance with the instructions of the manufacturer (Promega Corporation, Madison, WI, USA).

### Western blotting

SSM1, SSM2, SSM3, and NMuMG cells were lysed in RIPA buffer (R0278; Sigma-Aldrich) with 2 mM sodium vanadate, protease inhibitor cocktail (P8340, 1:500; Sigma-Aldrich), and phosphatase inhibitor cocktail (P5726, 1:100; Sigma-Aldrich). Cleared lysate (200 μg) was resolved on SDS-PAGE and transferred onto nitrocellulose membranes. ERα expression was detected by incubating blots with the monoclonal antibody 6F11 against ERα (VP-E613, 1:500; Vector Laboratories, Burlingame, CA, USA). PR expression was examined with a PR antibody against the C terminus of both PR-A and PR-B (sc-538, 1:500; Santa Cruz Biotechnology, Inc.).

### Ovariectomy and tumor transplantation

Female WT or STAT1^-/- ^mice were either sham-operated or ovariectomized at 6 to 8 weeks of age under general anesthesia. Two weeks after the surgery, 10^5 ^SSM1, SSM2, or SSM3 mammary tumor cells in 10 μL were injected into the inguinal fat pads of the mice. Alternatively, tumor fragments of about 1 mm in size were transplanted into the inguinal fat pads. Tumor growth was monitored by palpation once every 3 to 6 days and measured at two perpendicular diameters. The average of the two perpendicular measurements was plotted. In experiments in which exogenous E2 was supplemented to ovariectomized mice, 60-day release E2 pellets at 0.5 mg per pellet were used (Innovative Research of America, Sarasota, FL, USA). In the endocrine treatment experiment, nu/nu mice or STAT1^-/- ^mice were transplanted with 10^6 ^SSM3 tumor cells or tumor fragments, respectively. When the established tumors reached 5 mm in diameter, the animals were either sham-operated or ovariectomized. Tumor growth was monitored as described above.

### Immunophenotypic analyses on STAT1^-/- ^mammary glands

Mammary glands were harvested from 10- to 14-month-old STAT1^-/- ^female mice and digested in collagenase and hyaluronidase solution, as described in [31]. Single-cell suspension was prepared after dissociated tissues were treated with trypsin/DNase for 1 minute and dispase/DNase for 2 minutes and passed through 40-μm cell strainers. Cells were first blocked with anti-CD16 and anti-CD32 Fcγ receptors and normal rat serum and then stained with anti-TER119-PE/Cy7 (BioLegend, San Diego, CA, USA), anti-CD31-PE/Cy7 (BioLegend), anti-CD45-PE/Cy7 (BioLegend), anti-CD24-APC (BioLegend), or anti-CD49f-biotin (BioLegend) for 20 minutes at 4°C. Streptavidin-APC/Cy7 (BioLegend) was added, and cells were incubated for 20 minutes at 4°C. Stained cells were collected by using an LSRII flow cytometer (BD Biosciences, San Jose, CA, USA). Dead cells were gated out by using DAPI (Invitrogen Corporation). Cells depleted of CD31, CD45, and TER119 were further analyzed on the basis of their CD49f and CD24 surface expression. Myoepithelial cells were defined as CD49f^hi ^CD24^int^, whereas luminal epithelial cells were CD49f^int ^CD24^hi^, as established previously [31-33]. Flow cytometry profiles were analyzed by using FloJo software (TreeStar Inc., Ashland, OR, USA).

### STAT1 reconstitution

Retrovirus expressing GFP alone, STAT1.IRES.GFP, and STAT1 mutants Y701F.IRES.GFP and S727A.IRES.GFP was prepared by co-transfecting Phoenix cells with the retrovirus plasmid and pCMV.VSVg by using FuGENE HD (Roche, Basel, Switzerland). Supernatant was harvested 48 and 72 hours after transfection and overlayed on NMuMG, SSM1, SSM2, or SSM3 cells in the presence of 8 μg/mL of polybrene for 6 to 8 hours. Infection was carried out for 2 consecutive days. The infected cells were harvested on day 2 after infection for Western blotting to confirm expression and on day 3 for flow cytometry to quantitate the percentages of cells undergoing early apoptosis. Apoptosis was measured by a flow cytometry-based annexin V-binding assay in accordance with the instructions of the manufacturer (BD Biosciences). Only the early apoptotic cells (annexin V-positive, 7AAD-negative) were analyzed.

### Gene expression profiling analysis

Total RNAs were isolated from normal mammary glands of primary STAT1^-/- ^mammary tumors by using Trizol in accordance with the procedure of the manufacturer (Invitrogen Corporation). RNA integrity was confirmed by using an Agilent bioanalyzer (Agilent Technologies, Inc., Santa Clara, CA, USA). Labeled target cRNAs were synthesized and hybridized to Affymetrix GeneChip MOE 430 2.0 arrays in accordance with the instructions of the manufacturer. Raw data were modeled and normalized by using dChip [34]. Well-annotated mouse and human orthologs were identified by using the Mouse Genome Informatics database [35], and medians were used in cases of redundant probesets. The Herschkowitz data matrix contains 232 human breast cancer datasets and 122 murine datasets from 13 different mammary tumor models, which were analyzed by using 106 intrinsic genes common to the two species [36]. To combine our STAT1^-/- ^mammary tumor datasets with the Herschkowitz datasets, 96 of the 106 intrinsic genes were used because of platform differences. Gene-wise normalization was carried out separately with each dataset such that each gene has median zero and unit standard deviation. Distance-weighted discrimination (DWD) was used to merge our datasets with the Herschkowitz datasets to eliminate large systematic biases arising from different RNA purification procedures and distinct microarray platforms [37,38]. For unsupervised cluster analysis, the average linkage hierarchical clustering algorithm was then applied to the merged datasets by using XCluster [39] with the centered correlation coefficient as the similarity/dissimilarity metric. The gene expression heatmap and dendrogram were generated in Java TreeView [40] to visualize the relationship between the human breast cancer subtypes and murine mammary tumor models according to the gene expression intensities of the 96 genes. Gene expression profiling data have been deposited in the National Center for Biotechnology Information's Gene Expression Omnibus (GEO) under accession number GEO:GSE31942 [41].

### sigClust

sigClust examines the significance of a given clustering by testing the null hypothesis that the datasets are from the same Gaussian distribution [42]. We applied sigClust to five STAT1^-/- ^primary tumor samples and 63 human luminal breast cancer samples by using the 96-gene intrinsic gene list. The resulting *P *value is 0.99998, which indicates that the STAT1^-/- ^mammary tumors are highly likely to be in the same cluster with the human luminal breast cancer datasets. In addition, we applied sigClust on the MMTV_Neu and MMTV_PyMT datasets from the Herschkowitz study [36]. To avoid potential bias due to different sample sizes, we drew a set of five samples out of these two sets of data through 200 iterations and implemented the test on the set of five samples and the human luminal breast cancer datasets. The average *P *values were 0.9110 (MMTV_Neu), 0.9630 (MMTV_PyMT), and 0.9665 (both mouse models combined). These results indicate that the STAT1^-/- ^mammary tumor model exhibits a higher degree of resemblance to human luminal breast cancers at the gene expression level than the MMTV-Neu and MMTV-PyMT models do.

### Consensus clustering

To investigate the stability of clustering between STAT1^-/- ^mammary tumors and human luminal breast cancer, consensus clustering, which is a re-sampling-based technique that uses perturbation to simulate a set of new samples from the original merged dataset, was employed [43]. Consensus index on empirical clustering results across all perturbed datasets was then summarized by the normalized proportion of times that two samples were assigned to the same cluster. The underlying assumption is that the induced cluster composition is more trustworthy if the clustering is robust to sampling variability. We re-sampled 1,000 times and considered the number of clusters ranging from two to 15 for assessment. Using the 96-gene intrinsic gene list, we found that the five STAT1^-/- ^mammary tumors belong to the same cluster 95% of the time after 1,000 re-samplings with an interquartile range (IQR) of 0.9128 to 0.9775 (range = 0.8964 to 1.0), which indicates that the STAT1^-/- ^mammary tumors are molecularly homogeneous. The STAT1^-/- ^mammary tumors and human luminal breast cancers cluster together 62% out of 1,000 re-samplings with an IQR of 0.57 to 0.67 (range = 0.32 to 0.78). In contrast, the MMTV-Neu and MMTV-PyMT mouse models cluster with the human luminal breast cancer datasets only 42% out of 1,000 re-samplings with an IQR of 0.44 to 0.48 (range = 0.01 to0.64). These results further demonstrate that the molecular signature of the STAT1^-/- ^mammary tumors significantly overlaps with that of human luminal breast cancers.

### Statistical analyses

Time to onset was analyzed by the Kaplan-Meier product limit method, which generated the Kaplan-Meier survival curves. *P *values were reported by log-rank test. All numerical results are presented as mean and standard error of mean and represent data from a minimum of three independent experiments unless otherwise stated. Tumor growth curves were analyzed by a distribution-free test [44]. The unpaired *t *test for two independent samples was used to determine the statistical significance between the experimental groups and control groups. Wilcoxon signed rank test was used to compare STAT1 intensity levels in tumor samples and adjacent normal breast tissues. Association between clinicopathological characteristics and ERα status was tested by chi-squared test or Fisher exact test, whichever was appropriate. All tests were two-sided, and a *P *value of not more than 0.05 was considered significant. GraphPad Prism (GraphPad Software, Inc., La Jolla, CA, USA), SAS 9.2 (SAS Institute Inc., Cary, NC, USA), and R 2.11.1 [45] were used for all statistical analyses.

## Results

### A subset of human breast cancers display reduced STAT1 expression in neoplastic cells

To explore the role of STAT1 in breast tumor development, the relative cellular levels of STAT1 protein were immunohistochemically assessed in a cohort of 161 primary breast cancer samples (78 ERα^- ^and 83 ERα^+ ^cases) (Table 1) by using a STAT1-specific polyclonal IgG antibody that recognizes a STAT1 epitope shared by the human and mouse proteins (Figure 1A). Normal breast tissues from 11 patients with cancer and five healthy individuals were used as nontransformed controls (Table 2). STAT1 expression in epithelial cells and infiltrating stromal cells was quantified on the basis of the percentage of STAT1^+ ^cells (percentage score) and the intensity of the positive signal by using a three-tiered scale (intensity score) (Figure 1B and Materials and methods).

**Table 2:** Summary of the STAT1 staining results on normal breast tissues and paired breast tumors

		Normal breast tissues			Breast tumors		
Case	ERα^a^	Percentage of STAT1^+ ^epithelial cells^b^	STAT1 staining intensity^c^	STAT1 score^d^	Percentage of STAT1^+ ^neoplastic cells^b^	STAT1 staining intensity^c^	STAT1 score^d^
HN1	NA	4	2	6	NA	NA	NA
HN2	NA	4	1	5	NA	NA	NA
HN3	NA	4	2	6	NA	NA	NA
HN4	NA	3	1	4	NA	NA	NA
HN5	NA	4	2	6	NA	NA	NA
87	+	4	2	6	1	1	2
160	+	4	2	6	1	1	2
84	+	4	2	6	1	1	2
147	+	4	2	6	1	1	2
80	+	4	2	6	1	1	2
115	+	4	2	6	1	1	2
161	+	3	2	5	1	1	2
58	-	3	1	4	1	1	2
20	-	3	1	4	1	1	2
53	-	4	2	6	1	2	3
47	-	4	2	6	1	2	3

Normal breast tissues from healthy individuals: HN1, HN2, HN3, HN4, and HN5. Paired tumor samples with adjacent normal breast tissues: case 87, 160, 84, 147, 80, 115, 161, 58, 20, 53, and 47. ^a^Estrogen receptor-alpha (ERα) expression in breast tumors. ^b^Percentage score: 1, < 5%; 2, 5%-25%; 3, 26%-75%; 4, > 75%. ^c^Intensity score: 1 = low, 2 = intermediate, 3 = high. ^d^STAT1 score = percentage score + intensity score. +, positive; -, negative; NA, not applicable.

**Figure 2:**
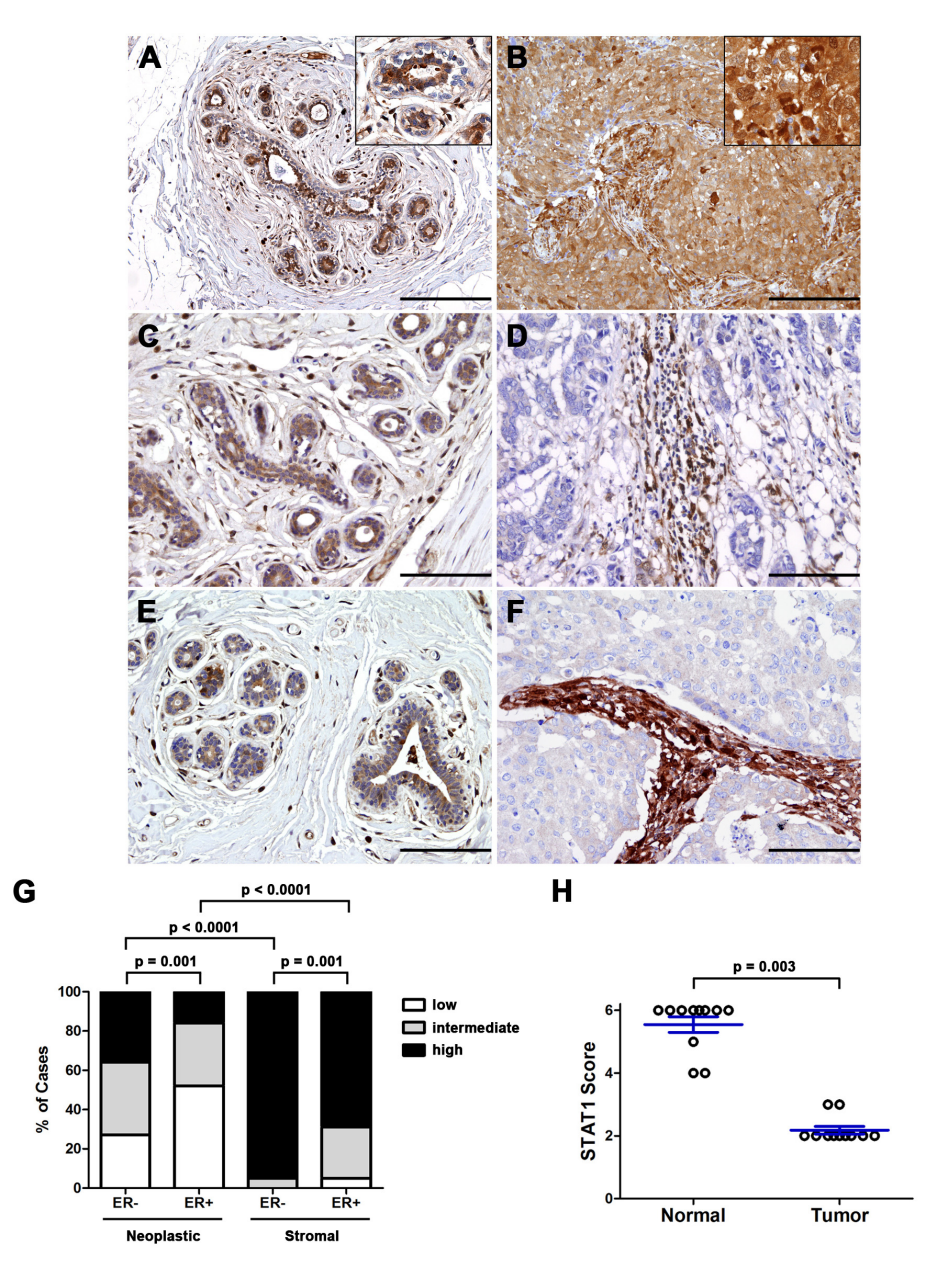
**Selective downregulation of STAT1 expression in the neoplastic cells of human breast tumors**. **(A) **Representative image of STAT1 staining on normal breast tissues of healthy individuals (*n *= 5) shows STAT1 expression in luminal epithelial cells and stromal cells and occasionally in myoepithelial cells. Original magnification, 100×. Scale bar = 200 μm. Inset, 400×. **(B) **Representative image of a breast tumor case with high levels of STAT1 expression in both the epithelial and stromal compartments. Original magnification, 200×. Scale bar = 100 μm. Inset, 400×. **(C-F) **Representative images of paired adjacent normal breast tissues (left) with breast tumors (right; *n *= 11). Morphologically normal breast tissues from patients with breast cancer display a STAT1 expression pattern that is similar to that in normal breasts from healthy individuals (C, E). Panel (C) is the paired normal tissue of the tumor in panel (D) (case 87), whereas panel (E) is the paired normal tissue of the tumor in panel (F) (case 58). The epithelial tumor cells of the representative estrogen receptor-alpha-positive (ERα^+^) (D) and ERα^- ^(F) breast cancers were devoid of STAT1 expression, whereas STAT1 level remained high in the tumor stroma. Original magnification, 200×. Scale bars = 100 μm. **(G) **Intensity scores of the neoplastic and stromal cells of all cases (*n *= 161) are plotted. Reduced STAT1 staining intensity was preferentially associated with ERα^+ ^human breast cancers relative to ERα^- ^breast cancers in both the neoplastic and stromal compartments (*P *= 0.001, unpaired *t *test). Stromal cells had an overall higher STAT1 expression level than the neoplastic cells of the same tumors (*P *< 0.0001, Wilcoxon signed rank test). **(H) **Breast tumors exhibited significant reduction in STAT1 score in comparison with matched normal breast tissues (*n *= 11) (*P *= 0.003, Wilcoxon signed rank test). STAT1 score is the sum of the score representing the percentages of STAT1^+ ^cells (percentage score) and that representing the STAT1 staining intensity (intensity score).

In normal human breast tissue from healthy individuals, STAT1 was detected in the cytoplasm of luminal epithelial cells, in the nuclei of the spindle cells in the surrounding stroma, and sporadically in myoepithelial cells (Figure 2A). In contrast, STAT1 expression was highly variable among tumor samples (Figures 1B, 2B, D, and 2F), consistent with previous reports [46,47]. Specifically, in 11% of cases (17 out of 161), STAT1 expression was detectable in less than 5% of the neoplastic cells, whereas in 37% of cases (59 out of 161), more than 75% of the tumor cells expressed STAT1 (percentage score in Table 1). The remaining 52% of cases displayed an intermediate phenotype in which STAT1 staining was observed in 5% to 75% of the neoplastic cells. In addition to documenting the percentage of STAT1^+ ^cells, we recorded the relative intensity of the positive signal (intensity score). Thirty-four percent of the tumor cases (54 out of 161) exhibited low STAT1 staining in the neoplastic cells, whereas 37% and 29% showed intermediate and high staining, respectively (Table 1). Although the percentages of STAT1^+ ^neoplastic cells in ERα^- ^and ERα^+ ^tumors were comparable (percentage score in Table 1), the intensity of the staining was significantly lower in ERα^+ ^breast cancers than in ERα^- ^breast cancers (intensity score in Table 1 and summarized in Figure 2G). Specifically, 45% of the ERα^+ ^tumors exhibited low levels of STAT1 staining in neoplastic cells, in contrast to 22% of the ERα^- ^cases, demonstrating that reduced STAT1 expression is associated with ERα^+ ^tumors (Table 1 and Figure 2G). A similar trend was observed in the stroma between ERα^- ^and ERα^+ ^cases (Figure 2G). However, since STAT1 was universally elevated in stromal cells compared with neoplastic cells within the same tumors (Figure 2D, F, and quantified in Figure 2G), the difference in stromal STAT1 intensity between ERα^- ^and ERα^+ ^cases is unlikely to be functionally meaningful. These findings thus indicate that STAT1 expression is differentially regulated in epithelial versus stromal compartments.

In contrast to the observation that lower STAT1 expression levels were associated with ERα^+ ^breast cancers versus ERα^- ^cancers, there was no significant association between low STAT1 expression and HER2 status (Table 3). HER2^- ^and HER2^+ ^breast cancers displayed similar percentages of STAT1^+ ^cells and levels of STAT1 intensity in the stromal and neoplastic compartments (Table 3). When these data are taken together, the lowest level of STAT1 expression is preferentially associated with the ERα^+ ^breast cancer subtype.

**Table 3:** Summary of the STAT1 staining results in the estrogen receptor-negative human breast cancer cohort stratified by HER2 status

	Number (percentage)			
	All ER^- ^cases	HER2^-^	HER2^+^	
Characteristics	(*n *= 78)	(*n *= 46)	(*n *= 32)	*P *value
Percentage of STAT1^+ ^neoplastic cells (percentage score)				
< 5%	7 (9%)	4 (9%)	3 (10%)	
5%-25%	10 (13%)	8 (17%)	2 (6%)	0.14^a^
26%-75%	33 (42%)	22 (48%)	11 (34%)	
> 75%	28 (36%)	12 (26%)	16 (50%)	
Percentage of STAT1^+ ^stromal cells (percentage score)				
< 5%	0 (0%)	0 (0%)	0 (0%)	
5%-25%	2 (3%)	2 (4%)	0 (0%)	0.2^a^
26%-75%	26 (33%)	18 (39%)	8 (25%)	
> 75%	50 (64%)	26 (57%)	24 (75%)	
STAT1 intensity in STAT1^+ ^neoplastic cells (intensity score)				
Low	17 (22%)	10 (22%)	7 (22%)	
Intermediate	31 (40%)	19 (41%)	12 (38%)	0.96^b^
High	30 (38%)	17 (37%)	13 (40%)	
STAT1 intensity in STAT1^+ ^stromal cells (intensity score)				
Low	0 (0%)	0 (0%)	0 (0%)	
Intermediate	3 (4%)	0 (0%)	3 (10%)	0.07^a^
High	75 (96%)	46 (100%)	29 (90%)	

^a^Fisher exact test. ^b^Chi-squared test. ER, estrogen receptor; HER2, human epidermal growth factor receptor 2.

Since STAT1 is uniformly expressed in the luminal epithelial cells of breast tissues from normal healthy individuals, the observation that a large subset of breast cancer cases exhibit low levels of STAT1 expression suggests that STAT1 may be downregulated specifically in the neoplastic cells. To further investigate this possibility, STAT1 expression was also assessed in morphologically normal breast tissues of patients with breast cancer. In all of the 11 STAT1-low breast cancer cases in which adjacent normal breast tissues were available, normal breast tissues had significantly more STAT1^+ ^epithelial cells and exhibited stronger staining intensity than matched tumor tissues (Figure 2C-F and Table 2). This resulted in an overall higher STAT1 score in normal epithelial cells than in breast tumor cells (Figure 2H). These findings thus demonstrate that STAT1 expression is dramatically diminished or lost in a significant proportion of breast cancer cells during tumor progression.

### STAT1^-/- ^mice are highly susceptible to mammary tumor formation

**Figure 3:**
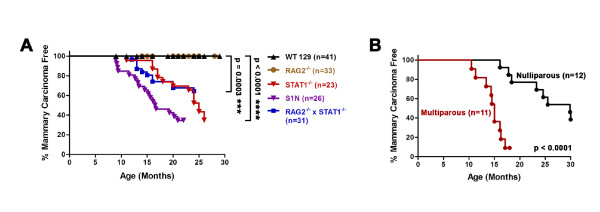
**Spontaneous development of mammary gland adenocarcinomas in STAT1 deficient (STAT1^-/-^), STAT1 null (S1N), and STAT1^-/- ^× RAG2^-/- ^female mice**. **(A) **STAT1^-/- ^(*n *= 23, inverted red triangles), S1N (*n *= 26, inverted purple triangles), and STAT1^-/- ^× RAG2^-/- ^(*n *= 31, blue squares) mice succumbed to mammary tumors, whereas none of the age-matched wild-type (WT) 129S6/SvEv (*n *= 41, black triangles) or RAG2^-/- ^(*n *= 33, yellow circles) mice developed the disease. *P *values were obtained with log-rank test by comparing STAT1^-/- ^× RAG2^-/- ^with WT mice (***P = 0.0003) and STAT1^-/- ^or S1N with WT mice (****P < 0.0001). Curves between STAT1^-/- ^and S1N mice are not different to a statistically significant degree (*P *= 0.24). **(B) **Multiparous STAT1^-/- ^mice (*n *= 11, red) developed mammary tumors at a higher frequency and with shorter latency than nulliparous STAT1^-/- ^mice (*n *= 12, black) (*P *< 0.0001).

The findings that diminished STAT1 expression is associated with breast cancer progression prompted us to investigate whether loss of STAT1 was a cause, not merely a consequence, of mammary tumorigenesis. We monitored WT and STAT1^-/- ^129S6/SvEv-strain female mice for tumor development and found that 65% (15 out of 23) of the STAT1^-/- ^mice developed spontaneous mammary adenocarcinomas (median tumor onset of 23 months) but that none of the WT mice developed the disease (Figure 3A). In agreement with our previous report [13], we observed mammary tumor development in a larger cohort of STAT1^-/- ^× RAG2^-/- ^female mice in similar disease incidences (Figure 3A). STAT1^-/- ^mice developed mammary tumors only, whereas STAT1^-/- ^× RAG2^-/- ^mice developed mammary and intestinal tumors. Mice lacking RAG2 alone did not develop mammary tumors (Figure 3A), demonstrating that this phenotype is specifically associated with STAT1 deficiency. Cells from our STAT1^-/- ^mice express low levels of a nonfunctional truncated STAT1 protein and are incapable of responding to IFN either *in vitro *or *in vivo *[10,48]. Nevertheless, by examining STAT1-null (S1N) mice generated by using a different targeting strategy and different embryonic stem cells that were maintained on a mixed C57BL/6-129/SvEv background, we ruled out the possibility that this truncated protein is involved in disease development [11]. Female S1N mice also developed spontaneous mammary tumors with a median tumor onset of 14.5 months (Figure 3A). Although the S1N mice exhibited a shorter latency than the 129S6/SvEv STAT1^-/- ^mice, the penetrance of the two cohorts was indistinguishable and the difference in the overall tumor incidences was not statistically significant (*P *= 0.26). These results demonstrate the generality of the phenotype to mice lacking functional STAT1, regardless of targeting strategy or mouse strain. STAT1^-/- ^mice that were sterilely re-derived and housed exclusively in a commercial gnotobiotic facility also developed mammary tumors, a result suggesting that the disease was not of infectious origin. This conclusion was substantiated by viral microarrays [49] that failed to detect either known or novel viruses in mammary tumors or other tissues of STAT1^-/- ^mice and by the lack of evidence for translocation and additional chromosomal integration of endogenous mammary tumor proviruses in these tumors (Figure S1 in Additional file 1).

Since tumor development did not reach a complete penetrance in the STAT1^-/- ^and S1N mice, we examined whether parity might influence tumorigenesis. Strikingly, multiparous STAT1^-/- ^mice developed mammary tumors sooner and at a higher frequency than nulliparous STAT1^-/- ^mice (Figure 3B). Specifically, multiparous mice had a median tumor onset of 14.8 months versus 24.6 months of nulliparous mice. Most importantly, the incidence of tumor development was 91% (10 out of 11) in mice that had undergone multiple rounds of pregnancy and lactation compared with 62% of nulliparous mice. Taken together, these data suggest that pregnancy-associated hormones might accelerate mammary tumor formation in STAT1^-/- ^mice.

### STAT1^-/- ^mammary tumors display homogeneous expression of estrogen receptor-alpha

**Figure 4:**
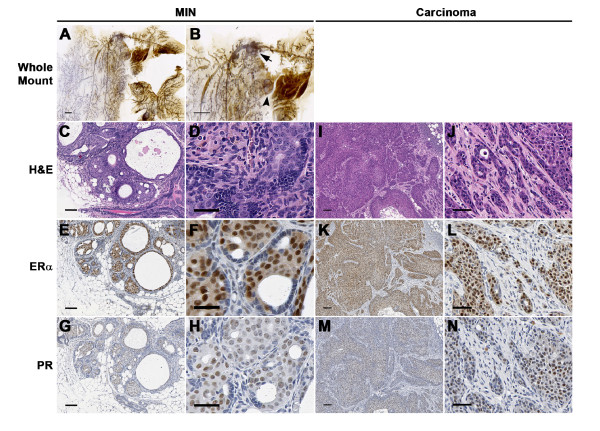
**Histopathological analyses of the mammary gland carcinomas developed in STAT1^-/- ^mice**. Histopathological analyses of the early lesions **(A-H) **and the invasive adenocarcinomas **(I-N) **in mammary glands of STAT1^-/- ^mice. (A, B) Whole-mount images of a thoracic mammary fat pad show aberrant dilation of mammary ducts and clusters of cystic alveoli (arrow and arrowhead). Panel (B) is a magnified image of panel (A). Scale bars = 1 mm. (C-H) Histology of the cluster of cysts shown in panel (B), highlighted by an arrow in panel (B), demonstrated mammary intraepithelial neoplasia (MIN). (C, D, I, J) Sections were stained with hematoxylin and eosin (H&E). Solid nests of neoplastic cells with areas of necrosis and invasion are evident in the carcinomas (I, J). (E-H, K-N) Atypical cells in MIN lesion and neoplastic cells expressed estrogen receptor-alpha (ERα) (E, F, K, L) and progesterone receptor (PR) (G, H, M, N). (C, E, G, I, K, M) Scale bars = 100 μm. (D, F, H, J, L, N) Higher magnification of the corresponding left panels. Scale bars = 40 μm.

Examination of whole mounts of mammary gland tissues from 12- to 24-month-old female STAT1^-/- ^mice without palpable masses revealed focal atypias in about 50% of the cohort (*n *= 19) (Figure 4A, B). This abnormality was not observed in mammary glands of age-matched WT female mice (*n *= 12). The early lesions in STAT1^-/- ^mammary glands varied from distended ducts to small cystically dilated clusters of alveoli (Figure 4B). Analysis of the H&E-stained tissue sections revealed that these abnormal foci contained atypical cells fulfilling the criteria for mammary intraepithelial neoplasia (MIN) (Figure 4C, D) [50]. Surprisingly, atypical nuclei in MIN lesions stained positively for ERα and PR (Figure 4E-H). Similar analyses of overt tumors revealed the presence of solid nests of neoplastic ERα^+ ^or PR^+ ^cells with frequent central necroses (Figure 4I-N). Over 90% of all tumor cells expressed ERα (Figure 4K, L). Some tumors had invasive nests of neoplastic cells with areas of fibrosis and inflammation (Figure 4I, J). All primary STAT1^-/- ^mammary tumors examined, regardless of parity, showed similar histopathological characteristics and patterns of hormone receptor expression. None of the primary tumors stained positively for HER2 (data not shown). Thus, mammary tumors in STAT1^-/- ^mice progress developmentally from intraepithelial neoplasia to carcinoma in a manner that is remarkably similar to the progression from ductal carcinoma *in situ *to invasive ductal carcinoma seen in the human disease [51]. Since spontaneous ERα^+^/PR^+ ^mammary tumors are rarely observed in mice, our findings also suggested that STAT1^-/- ^mice may represent a relatively novel model for human ERα^+^/PR^+ ^luminal breast cancer.

### Ovarian hormone dependency of STAT1^-/- ^mammary tumor cells

**Figure 5:**
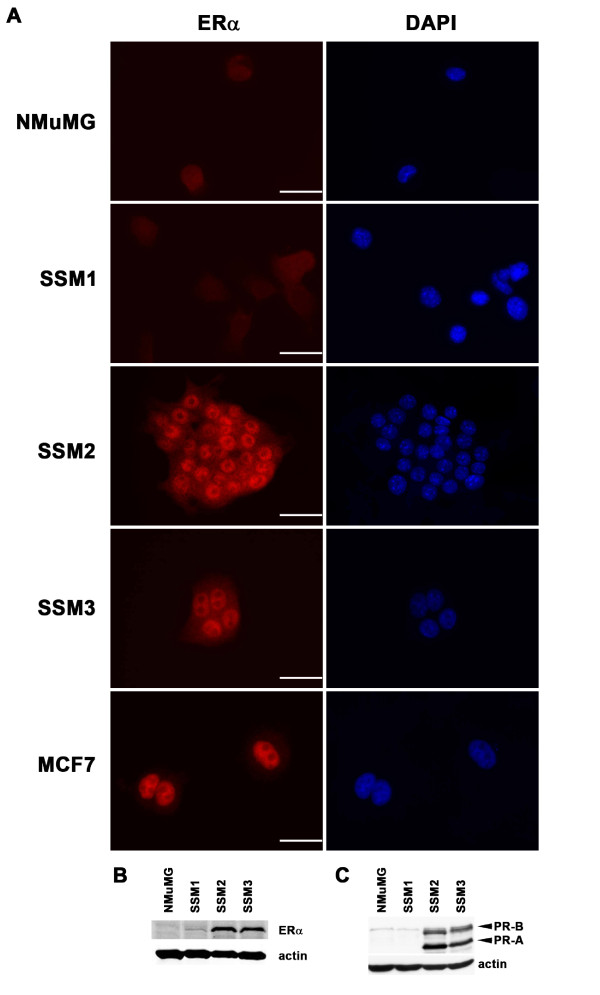
**STAT1^-/- ^mammary tumor cell lines SSM2 and SSM3 express estrogen receptor-alpha (ERα), PR-A, and PR-B**. **(A) **SSM2 and SSM3 expressed nuclear ERα, similar to the human ERα^+ ^breast cancer cell line MCF7. In contrast, NMuMG and SSM1 did not exhibit nuclear ERα staining. Scale bars = 40 μm. **(B) **SSM2 and SSM3 are positive for ERα expression, but SSM1 expressed a very low level of ERα, by Western blot analysis. NMuMG exhibited no detectable levels of ERα. **(C) **SSM2 and SSM3 expressed PR-A and PR-B, which are derived from alternative PR promoters and are target genes for ERα signaling, suggesting that SSM2 and SSM3 were estrogen-responsive. In contrast, NMuMG and SSM1 did not display functional ERα signaling. DAPI, 4'-6-diamidino-2-phenylindole; SSM, spontaneous STAT1^-/- ^mammary (epithelial tumor cell line).

**Figure 6:**
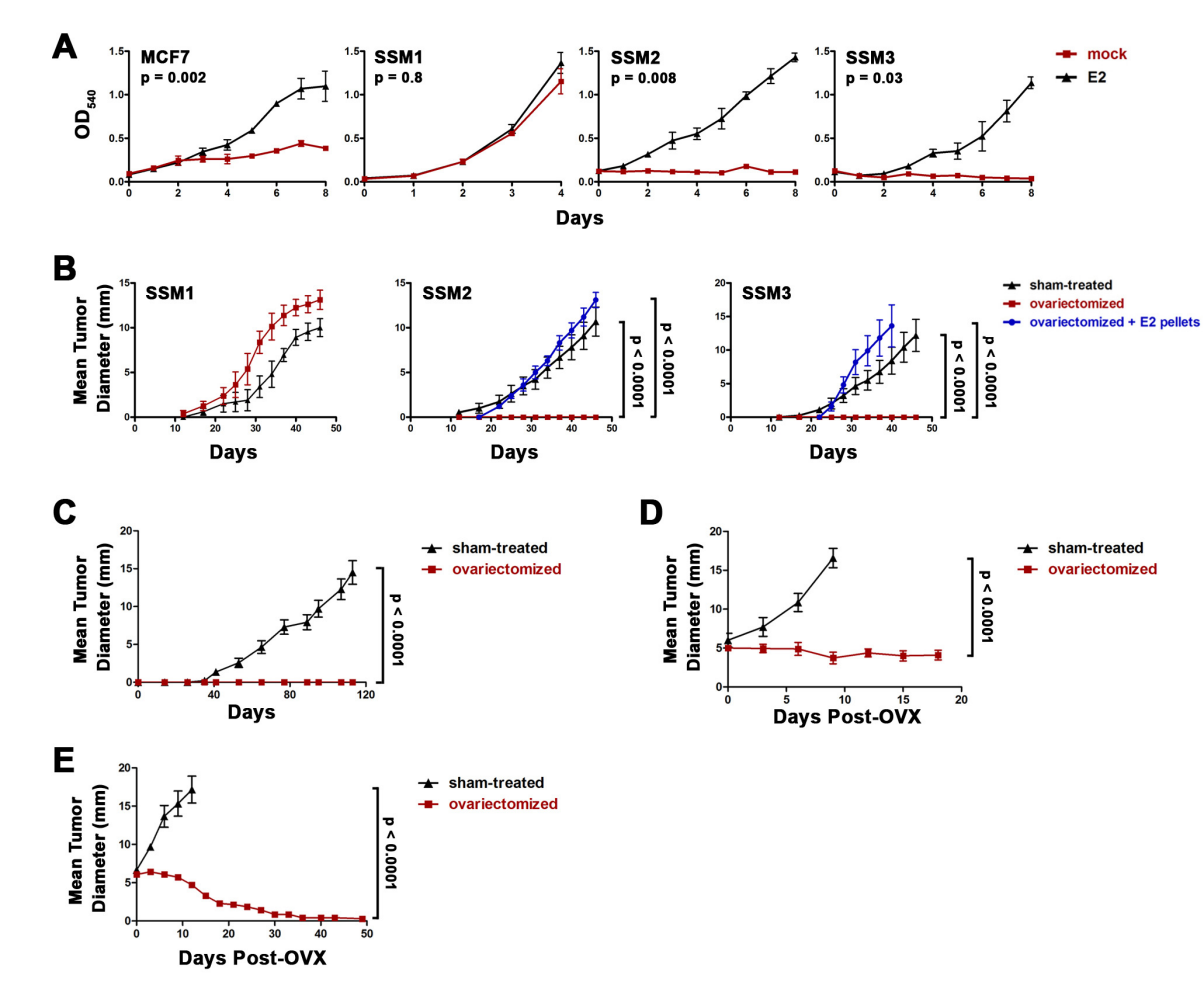
**STAT1^-/- ^mammary tumors respond to and depend on ovarian hormones for engraftment and tumor progression**. **(A) **SSM1, SSM2, SSM3, or MCF7 was plated in phenol red-free media containing charcoal-treated fetal calf serum in the presence (17β-estradiol, or E2; black triangles) or absence (mock; red squares) of 10 nM E2. *P *values were obtained with *t *test comparing mock-treated and E2-treated samples. **(B) **SSM1, SSM2, or SSM3 (10^5 ^cells per mouse) was transplanted into the inguinal mammary glands of sham-treated (black triangles), ovariectomized (red squares), or ovariectomized wild-type or STAT1^-/- ^mice that were supplemented with E2 pellets (blue circles). *P *values were obtained with *t *test comparing tumor growth in ovariectomized mice with that in either sham-treated or ovariectomized mice supplemented with E2 pellets [44]. **(C) **Growth of tumor transplants from primary STAT1^-/- ^mammary tumors is ovarian hormone-dependent. Fragments (1 × 1 mm^2^) from two primary STAT1^-/- ^mammary tumors were transplanted into the inguinal fat pads of ovariectomized mice (red) or sham-operated mice (black). No palpable masses were detected in ovariectomized mice. In contrast, tumors grew progressively in recipients with intact ovaries. Results represent seven to eight mice in each group (*P *< 0.0001). **(D) **SSM3 tumor cells were transplanted orthotopically into the mammary fat pads of nude mice. Mice bearing established tumors around 5 mm in diameter were sham-operated (black triangles; *n *= 7) or ovariectomized (red squares; *n *= 9). SSM3 tumors failed to progress in the absence of ovarian hormones, demonstrating sensitivity to ovarian hormone deprivation therapy (*P *< 0.0001). **(E) **Fragments of primary STAT1^-/- ^mammary tumors were transplanted into the mammary fat pads of STAT1^-/- ^mice. When the established tumors reached 5 mm in diameter, the animals were either sham-operated (black triangles; *n *= 3) or ovariectomized (red squares; *n *= 7). Transplanted primary tumors regressed after ovarian hormone deprivation (*P *< 0.0001). Error bars indicate standard error of the mean. OD_540_, optical density at 540 nm; OVX, ovariectomy; SSM, spontaneous STAT1^-/- ^mammary (epithelial tumor cell line).

To facilitate biological and biochemical characterization of the STAT1^-/- ^mammary tumors, three tumor cell lines were established and designated spontaneous STAT1^-/- ^mammary (SSM) epithelial tumor cell lines (SSM1, SSM2, and SSM3). All expressed CK but not vimentin *in vitro*, documenting their epithelial origin (Figure S2 in Additional file 2). SSM2 and SSM3 expressed nuclear ERα, which is similar to the human ERα^+ ^breast cancer cell line, MCF7 (Figure 5A). SSM2 and SSM3 also expressed the two PR isoforms, PR-A and PR-B (Figure 5C), suggesting that ERα is functional in these cells. In contrast, SSM1 expressed a very low level of ERα (Figure 5B) and no detectable PR-A/PR-B expression (Figure 5C), indicating a lack of ERα signaling in the SSM1 cells. Whereas SSM2 and SSM3 required the presence of estrogen to proliferate *in vitro*, SSM1 did not (Figure 6A). Finally, when transplanted into the fat pads of WT or STAT1^-/- ^mice, SSM2 and SSM3 grew only in recipients with intact ovaries or in ovariectomized recipients that received subcutaneous estrogen pellet implants (Figure 6B). In contrast, SSM1 did not require ovarian hormones for *in vivo *growth (Figure 6B), revealing that not all STAT1^-/- ^mammary tumor cells display hormone dependency. This result corresponds with the finding that most, but not all, tumor cells in the primary STAT1^-/- ^mammary carcinomas display ERα and PR positivity (Figure 4). However, these hormone-independent cells are extremely rare in the primary STAT1^-/- ^mammary tumors as demonstrated by the fact that tumor fragments from two independent primary tumors failed to engraft in ovariectomized mice (Figure 6C). Together, these results demonstrate that SSM2 and SSM3 are ovarian hormone-responsive and -dependent *in vitro *and *in vivo *and that SSM1 arises from a very rare subset of ERα^- ^cells and is used in this study as a hormone-nonresponsive cell line.

Having demonstrated that the STAT1^-/- ^ERα^+ ^mammary tumor cells required ovarian hormones for establishment of tumor growth, we next examined whether these tumors also depended on ovarian hormones to maintain tumor progression. We used ovariectomy as the treatment modality since ovarian ablation is commonly used to treat premenopausal patients with ERα^+^/PR^+ ^breast cancers [52]. Established SSM3 tumors continued to grow in sham-treated mice (Figure 6D). However, established tumors failed to progress after ovariectomy, indicating that the STAT1^-/- ^ERα^+^/PR^+ ^tumor cells require ovarian hormones not only for *in vivo *engraftment but also for maintenance of growth. Tumors that were established from transplanted primary tumor fragments were also highly sensitive to ovarian hormone deprivation and became unpalpable upon ovariectomy (Figure 6E). Thus, the STAT1^-/- ^mammary tumors are functionally similar to human ERα^+^/PR^+ ^breast cancers.

### STAT1^-/- ^mammary tumors display a surface marker phenotype reflective of luminal mammary tumors

**Figure 7:**
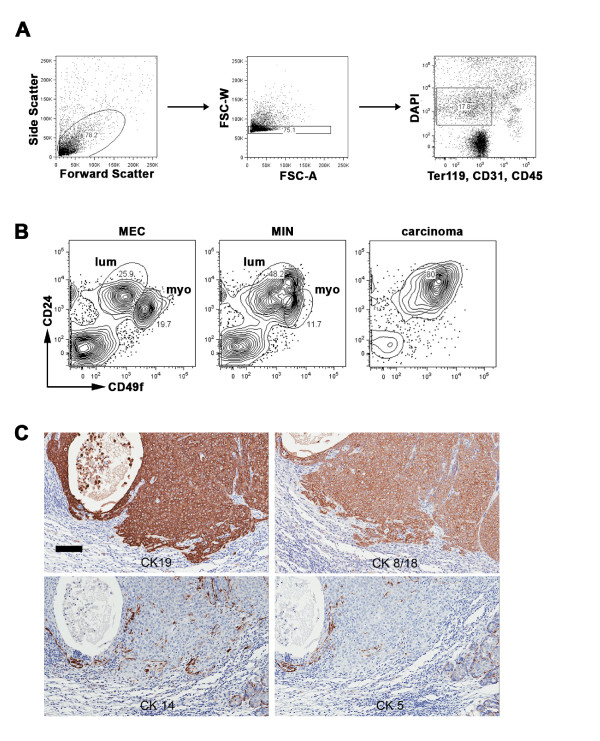
**STAT1^-/- ^mammary tumor cells display a luminal epithelial cell phenotype**. **(A) **Gating procedure used for the analysis of cell surface markers. Disaggregated mammary glands or mammary tumors were collected by an LSRII flow cytometer and analyzed by using FlowJo. Cells were first selected on the basis of size by using forward and side scatter (left panel). Single cells were then selected by forward scatter (FSC)-A and FSC-W (middle panel). Live cells (DAPI^-^) and lineage^- ^cells (Ter119^-^, CD31^-^, and CD45^-^) were gated for the analysis depicted in **(B)**. (B) Expression of CD49f and CD24 on STAT1^-/- ^mammary epithelial cells (MECs), mammary intraepithelial neoplasia (MIN), and carcinoma. Representative images from five STAT1^-/- ^mice are shown. Myoepithelial (myo) and luminal epithelial (lum) are highlighted. **(C) **Immunohistochemical analysis of primary STAT1^-/- ^mammary tumors for cytokeratin (CK) 5, 14, 8/18, and 19. Mammary tumor cells were stained positive for CK19 and CK8/18 (luminal epithelial markers) but negative for CK5 and CK14 (myoepithelial markers). Scale bar = 100 μm. DAPI, 4'-6-diamidino-2-phenylindole.

Hormone receptor expression is one of the critical parameters used to determine the suitable treatments for human patients with breast cancer [2]. In addition to hormone receptors, mammary tumors can be classified by biomarkers that are expressed on the tumor cell surface. Myoepithelial and luminal epithelial cells can be identified on the basis of the well-established differential expression of murine mammary epithelial cell population markers, CD49f and CD24 (that is, myoepithelial cells = CD49f^hi ^CD24^int ^and luminal epithelial cells = CD49f^int ^CD24^hi^) [31-33]. These two epithelial cell populations could be readily differentiated in nontransformed mammary glands of STAT1^-/- ^mice ('myo' and 'lum' in Figure 7B) by flow cytometry upon elimination of dead cells and lineage-positive cells (Figure 7A). Marked expansion of the luminal epithelial cell subset was evident in MIN, the earliest stage of neoplastic changes identifiable by histology, in comparison with nontransformed mammary glands (Figure 7B). Further expansion of these luminal epithelial cells continued as MIN progressed to carcinomas (Figure 7B). Neoplastic cells in carcinomas homogeneously displayed a CD49f^int ^CD24^hi ^phenotype. Consistent with this result, primary STAT1^-/- ^mammary tumors are strongly positive for the luminal epithelial markers, CK19 and CK8/18 (Figure 7C). Occasional CK5^+^, CK14^+^, or p63^+ ^myoepithelial cells were observed (Figure 7C and data not shown). Together, these results indicate that the STAT1^-/- ^mammary tumor cells exhibit a marker phenotype that is characteristic of luminal epithelial cells.

### STAT1^-/- ^mammary tumors exhibit human luminal breast cancer-like molecular signatures

**Figure 8:**
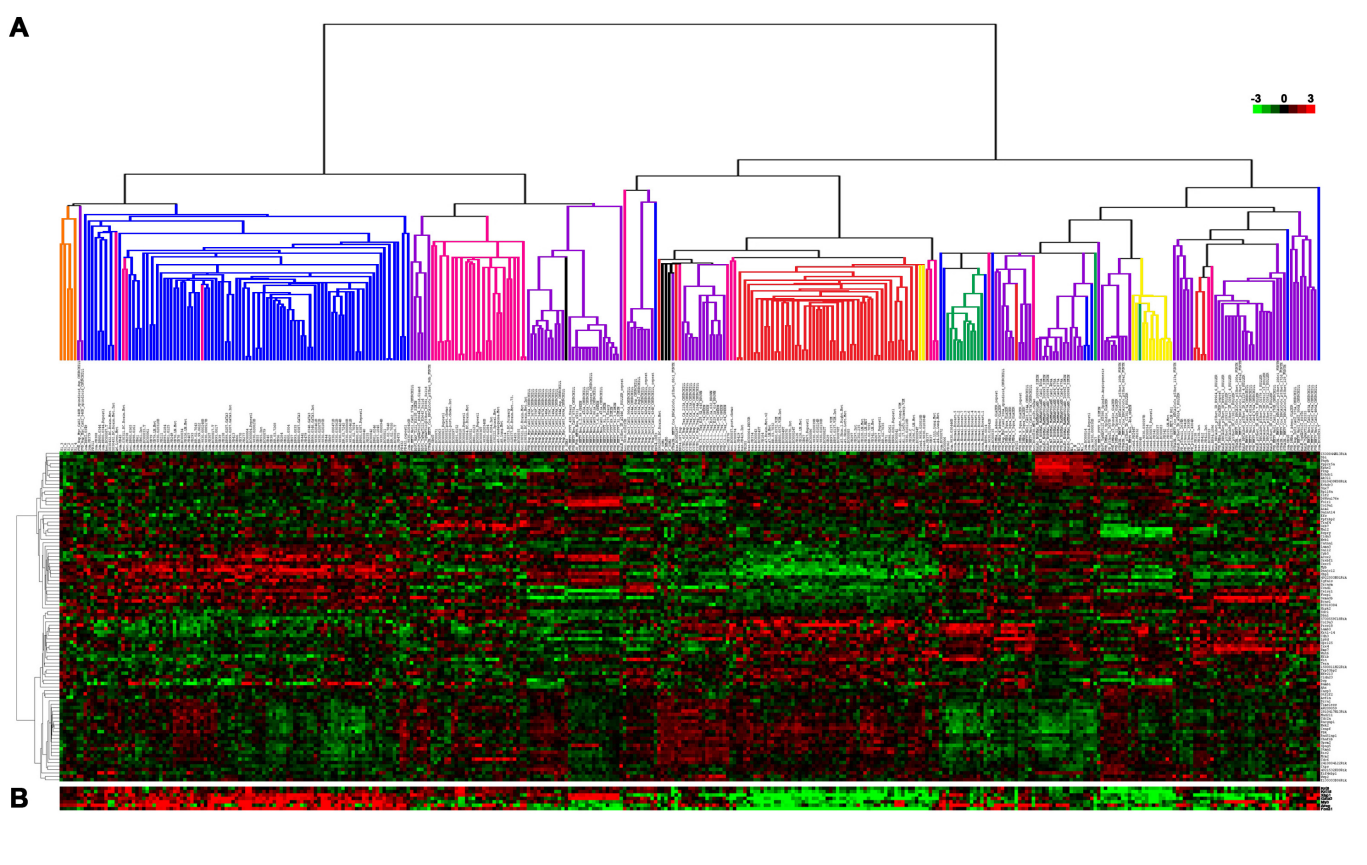
**Gene expression profiles of primary STAT1^-/- ^estrogen receptor-alpha-positive (ERα^**+**^) mammary tumors show significant overlap with that of human ERα^**+ **^luminal breast cancers**. **(A) **Five primary STAT1^-/- ^ERα^+ ^mammary tumor datasets (S1_1, S1_2, S1_3, S1_4, and S1_6; orange) were analyzed with 232 annotated human breast cancer datasets and 13 other mouse mammary tumor models (purple) by hierarchical clustering using 96 genes known to be among those that are diagnostic for different human breast cancer subtypes [36]. Genes are represented in rows, and datasets are represented in columns. Red represents genes that are overexpressed compared with the median, whereas green represents genes that are underexpressed compared with the median. The STAT1^-/- ^ERα^+ ^mammary tumors (orange) cluster closely to the human luminal breast cancers (blue) at the far left of the heatmap, underscoring the significant relatedness between the two cohorts. All human samples are colored by intrinsic subtype as determined in [36]: blue = luminal, red = basal-like, pink = HER2^+^/ER^-^, yellow = claudin-low, and gree*n *= normal breast-like. Expression values of the datasets can be found in Additional file 3. **(B) **Display of seven genes that are important identifiers for the luminal subtype: *KRT8*, *KRT18*, *XBP1*, *GATA3*, *MYB*, *AREG*, and *FOXA1 *(from top to bottom). See Additional file 3 for the expression values of these genes. HER2, human epidermal growth factor receptor 2.

Perou and colleagues [3,53] reported previously that human breast cancers could be differentiated into five molecular subtypes (luminal A, luminal B, HER2, basal, and normal-like) on the basis of their gene expression profiles. Strikingly, when gene expression patterns of mammary tumors from 13 different pre-existing mouse breast cancer models were compared with those of 232 human breast cancers, none showed significant overlap with human ERα^+ ^luminal breast cancers [36]. To extend these findings, we compared the gene expression patterns of the aforementioned datasets (primary data generously provided by Dr. Charles M Perou, University of North Carolina at Chapel Hill) with those of five primary STAT1^-/- ^ERα^+ ^mammary tumors. Hierarchical clustering analysis revealed that the five primary STAT1^-/- ^ERα^+ ^mammary tumors not only resembled one another but also closely resembled human luminal breast cancers (Figure 8A). STAT1^-/- ^ERα^+ ^mammary tumors also showed elevated transcription of genes characteristic of luminal breast cancers (for example, *keratin 8*, *keratin 18*, *XBP1*, *GATA3*, *MYB*, *AREG*, and *FOXA1*) (Figure 8B). In agreement with the previous report [36], samples from other mouse mammary tumor models consistently clustered away from human luminal breast cancers (Figure 8A). Finally, analyses using two other statistical methods (sigClust [42] and consensus clustering [43]) also supported the conclusion that STAT1^-/- ^ERα^+ ^mammary tumors grouped more consistently and reproducibly with human luminal breast cancers than any other mouse mammary tumor model (see Materials and methods). Specifically, results from the consensus clustering analysis indicated that the STAT1^-/- ^mammary tumors and human luminal breast cancers clustered 62% of the time upon 1,000 re-samplings but that the MMTV-Neu and MMTV-PyMT clustered with human luminal breast cancers only 42% of the time upon 1,000 re-samplings. Together, the STAT1^-/- ^ERα^+ ^mammary tumors display high molecular homogeneity and a striking similarity to human luminal breast cancers.

### A cell-autonomous role of STAT1 in suppressing mammary tumor formation

**Figure 9:**
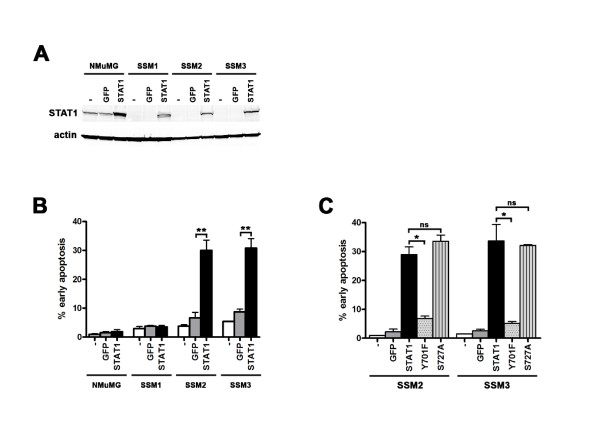
**Tumor suppressor function of STAT1 is cell-autonomous**. **(A) **STAT1 was ectopically expressed in SSM1, SSM2, and SSM3 by retroviral transduction (STAT1) to levels comparable to the endogenous level in nontransduced (-) NMuMG. STAT1 was also overexpressed in NMuMG (STAT1). Retrovirus expressing GFP alone was used as a negative control (GFP). **(B) **STAT1 reconstitution in SSM2 and SSM3 resulted in 4.5- and 3.5-fold increases in early apoptosis (that is, annexin V-positive, 7-AAD-negative cells) 3 days post-transduction, respectively. **P < 0.005. **(C) **Tyrosine 701 is required for STAT1-mediated apoptosis in SSM2 and SSM3. Retrovirus expressing GFP, WT STAT1 (STAT1), and STAT1 mutants (Y701F or S727A) were transduced into SSM2 and SSM3. Mutation in Y701 (Y701F) abolished the ability of STAT1 to induce cell death, whereas that in S727 (S727A) was still capable of inducing cell death. SSM2, *P = 0.02. SSM3, *P = 0.04. *P *values were obtained with unpaired *t *test. ns, not significant; SSM, spontaneous STAT1^-/- ^mammary (epithelial tumor cell line).

The above observations led us to conclude that STAT1 suppresses tumor development in the mammary gland. It is possible that STAT1 confers this role by mediating the elimination phase of immunoediting. However, since the immunodeficient RAG2^-/- ^mice never developed mammary tumors and the loss of STAT1 expression was observed only in the neoplastic cells of human breast cancers, we hypothesized that STAT1 might act as a cell-intrinsic tumor suppressor in mammary epithelium. We first investigated whether enforced expression of STAT1 in STAT1^-/- ^mammary tumor cells affected their tumorigenic phenotype. This approach has been used in the past to define several other tumor suppressors [54]. Retroviral transduction of WT STAT1 into SSM1, SSM2, and SSM3 resulted in expression of STAT1 protein levels in each cell line comparable to that in unmanipulated NMuMG (Figure 9A). Enforced expression of WT STAT1 led to apoptosis of a substantial percentage of SSM2 and SSM3 but not of SSM1 or NMuMG (Figure 9B). Therefore, the tumor suppression function of STAT1 is cell-autonomous.

Since phosphorylation at the tyrosine residue at position 701 synergizes with that at the serine residue at position 727 of STAT1 to effect the maximal transcriptional activity [53,55], two STAT1 mutants, one lacking the Tyr residue (Y701F) and one lacking the Ser residue (S727A), were tested for their ability to induce tumor cell death. Even when expressed at levels comparable to those achieved with WT STAT1, the Y701F mutant was unable to induce apoptosis when expressed in SSM2 and SSM3 (Figure 9C). In contrast, the S727A mutant displayed an indistinguishable phenotype as the WT STAT1. Thus, the tumor suppressor action of STAT1 requires tyrosine phosphorylation, but not serine phosphorylation, of STAT1.

## Discussion

Current dogma, based on gene expression analyses of intact breast cancer biopsies, holds that STAT1 mRNA levels are elevated in breast cancer tissues compared with normal breast tissues [3], leading to the hypothesis that STAT1 might facilitate tumor outgrowth. Here, we have presented a novel finding demonstrating a selective loss of STAT1 expression in neoplastic epithelial cells, but not in the surrounding stromal cells, compared with normal mammary epithelium. Additionally, this tumor cell-specific effect was observed more frequently in ERα^+ ^than in ERα^- ^human breast cancers. Therefore, an increase in STAT1 mRNA levels in the subset of breast cancer cases that exhibit low STAT1 expression in the neoplastic cells could be explained by a selective upregulation of STAT1 transcription in the stromal cells alone. Our findings thus indicate that the regulation of STAT1 expression is cell context-dependent and a STAT1 activation signature in whole-tumor biopsy might not reflect the biology of the entire tumor microenvironment. The clinical implication of these findings is that caution should be taken in interpreting the involvement of STAT1 in treatment outcomes when STAT1 activation signature in whole-tumor biopsies is used as a prognostic indicator.

Since downregulation of STAT1 expression is restricted to the neoplastic epithelial cells but not to the surrounding stromal cells, somatic silencing of STAT1 transcription may occur preferentially in the breast cancer cells. STAT1 promoter methylation in squamous cell carcinomas and prostate cancers has been proposed to be a mechanism whereby STAT1 transcription is repressed during transformation [56,57]. It is conceivable that STAT1 promoter methylation is likewise in action during breast cancer progression. However, we cannot eliminate the possibility that STAT1 downregulation occurs at the post-transcriptional level. Investigation undertaken to differentiate these possibilities and to determine whether STAT1 loss correlates with clinical outcome is ongoing.

To investigate the role of STAT1 in mammary tumorigenesis, we employed a novel murine system that provided mechanistic insights into the physiological consequence of loss of STAT1 expression. Specifically, we find, much to our surprise, that STAT1^-/- ^female mice spontaneously develop mammary gland adenocarcinomas that show remarkable similarities to human ERα^+ ^luminal breast cancers. Pathologically, the STAT1^-/- ^mammary tumors progress from a preneoplastic state classified as mammary intraepithelial neoplasia to adenocarcinoma, mirroring the progression of human breast cancer from atypical hyperplasia to ductal carcinoma *in situ *and finally to invasive carcinoma. The hormone receptor status of the STAT1^-/- ^mammary tumors also shows a remarkable parallel to human ERα^+^/PR^+ ^breast cancers. Biologically, STAT1^-/- ^mammary tumor cells depend on ovarian hormones for both the initiation and the maintenance of tumor growth. CD49f^int ^CD24^hi ^luminal epithelial cells are enriched for hormone receptor-positive cells that are rarely found in the myoepithelial cell subset [58]. It is tempting to speculate that the tumor-initiating cells in the STAT1^-/- ^mammary tumors reside in the luminal epithelial subset because of the significant expansion of these cells in the preneoplastic lesions. Future work will be focused on elucidating the nature of these tumor-initiating cells.

The penetrance of multiparous STAT1^-/- ^mice is remarkably close to 100%, suggesting that pregnancy-associated hormones can accelerate tumorigenesis. At present, it is not possible to conclude that these hormones are required for the tumorigenesis of the STAT1^-/- ^mammary glands since nulliparous STAT1^-/- ^mice also develop mammary tumors. While elucidating the roles of pregnancy-associated hormones in mammary tumorigenesis will be the target for future investigation, work investigating the mechanism by which STAT1 suppresses tumor formation has begun. We employed a classic approach that has been used to validate tumor suppressors in the past [54]. Similar to the classic tumor suppressors, restoration of WT STAT1 in the STAT1^-/- ^mammary tumor cells spontaneously causes tumor cell death. Our findings thus demonstrate that the tumor suppressor function of STAT1 is cell-autonomous. A mutant form of STAT1 lacking the functionally critical Tyr 701 residue is defective in this function, suggesting that STAT1 suppresses tumor formation by regulating the transcription of its target genes. Since phosphorylation in Ser 727 is functionally distinct from and independent of that in Tyr 701 [55,59,60], the inability of the S727A mutant to abrogate cell death suggests that tumor suppression mediated by STAT1 does not require S727-dependent target genes, like *GBP-1 *[55]. Although we cannot completely eliminate the possibility that STAT1 can also act as an extrinsic tumor suppressor via its ability to mediate functional anti-tumor immunity, the cell-intrinsic effect of STAT1 is consistent with recent studies demonstrating a role for STAT1 in suppressing ErbB2/Neu-driven tumor formation [21,22]. Epithelial-specific deletion of STAT1 accelerates tumor development in the ErbB2/Neu tumor model. Therefore, STAT1 might exert a broader tumor suppression function against multiple oncogenic pathways. It is then noteworthy that STAT1 expression is also diminished in the neoplastic cells of 22% of the human HER2^+ ^breast cancer cases that we examined in this study.

Although the current repertoire of endocrine therapy is remarkably effective in treating ERα^+ ^breast cancers, about 30% to 50% of the patients still suffer from recurrences [61-63]. Novel therapeutic targets for the treatment of ERα^+ ^breast cancers are, therefore, still needed. Preclinical models of human ERα^+^/PR^+ ^breast cancers are essential for the testing of new treatments. However, only a limited number of models produce tumors that contain a significant proportion of hormone-dependent ERα^+^/PR^+ ^tumor cells [5-8]. In addition, very little is known about the molecular characteristics of the few existing mouse ERα^+^/PR^+ ^tumor cell lines and thus it has not been possible to establish their genetic relationship to human luminal breast cancers. In contrast, STAT1^-/- ^mammary tumors exhibit well-defined tumor progression kinetics and a set of highly reproducible and homogeneous histopathological, biological, and molecular characteristics that closely resemble human luminal breast cancers. Most importantly, STAT1^-/- ^mammary tumors express elevated levels of ERα, PR, GATA3, AREG, XBP1, and FOXA1, all of which are regulated by the transcriptional control of ERα. In agreement with this activated ERα genetic signature, STAT1^-/- ^mammary tumor is also a unique preclinical model because of its sensitivity to standard endocrine therapy, including estrogen deprivation therapy (this study) and treatment targeting ERα (AM Fowler and MJ Welch, manuscript in preparation). Furthermore, STAT1^-/- ^mammary tumor cells are transplantable orthotopically into both immunocompetent and immunodeficient mice, facilitating the examination of immune-based therapies, which otherwise would not be possible in xenograft models using ERα^+^/PR^+ ^human breast cancer cell lines. Thus, this model not only allows one to study the entire developmental program of luminal mammary tumorigenesis but also permits short-term experiments using a tumor cell transplantation approach. For these reasons, the STAT1^-/- ^mammary tumor is an exceptional model for human ERα^+ ^PR^+ ^luminal breast cancers.

## Conclusions

The important findings of this study are that loss of STAT1 expression is a frequent event during the progression of human breast cancers and that loss of functional STAT1 in mice causes spontaneous development of mammary adenocarcinomas. These murine STAT1^-/- ^mammary tumors closely recapitulate the progression and biology of human ERα^+ ^luminal breast cancers. This is underscored by the potent anti-tumor action of ovarian ablation therapy on the STAT1^-/- ^mammary tumors. Our results thus validate the physiological relevance of our novel mouse ERα^+^/PR^+ ^STAT1^-/- ^mammary tumors for potential translatability to human breast cancer research.

## Abbreviations

CK: cytokeratin; DAPI: 4'-6-diamidino-2-phenylindole; DMEM/F12: Dulbecco's modified Eagle's medium/F12; E2: estradiol; ERα: estrogen receptor-alpha; FBS: fetal bovine serum; H&E: hematoxylin and eosin; HER2: human epidermal growth factor receptor 2; IFN: interferon; IQR: interquartile range; MIN: mammary intraepithelial neoplasia; PBS: phosphate-buffered saline; PR: progesterone receptor; S1N: STAT1-null; Ser: serine; SSM: spontaneous STAT1^-/- ^mammary (epithelial tumor cell line); Tyr: tyrosine; WT: wild-type.

## Competing interests

The authors declare that they have no competing interests.

## Authors' contributions

SRC conceived and planned the study, analyzed data, wrote the paper, analyzed the results, monitored mammary tumor development in different cohorts of mice, generated the SSM cell lines, and performed all of the biochemical, molecular, and *in vivo *studies. RDS conceived and planned the study, analyzed data, and wrote the paper. SL performed immunohistochemical studies on the human breast cancer biopsies. WV and LL analyzed the results. DEL monitored mammary tumor development in different cohorts of mice. JL performed gene expression profiling analyses and oversaw all statistical analyses. LJTY performed whole-mount analyses. CR performed Southern blotting. CA performed molecular studies. AMF and MJW contributed to study design. RDC analyzed the pathology of the STAT1^-/- ^mammary tumors. All authors read and approved the final manuscript.

## Supplementary Material

Additional file 1 **Supplementary Figure **1. **Evidence against translocation of endogenous murine mammary tumor virus (MMTV) as the cause of STAT1^-/- ^mammary tumorigenesis**. Southern blot analysis was used to detect the translocation of MMTV long terminal repeats (LTR). Genomic DNA was harvested from nontransformed mammary glands of young or aged nulliparous STAT1^-/- ^mice (lanes 2, 3, 12, and 13), nontransformed mammary glands of retired STAT1^-/- ^breeders (lanes 4 and 14), primary STAT1^-/- ^mammary tumors (lanes 6, 7, 16, and 17), or SSM cell lines established from primary STAT1^-/- ^mammary tumors (lanes 8 to 10 and lanes 18 to 20) and digested with either EcoRI (lanes 1 to 10) or PvuII (lanes 11 to 20). The number and the sizes of the DNA fragments hybridized to the MMTV LTR in these samples are indistinguishable from those in WT mammary glands (lanes 1 and 11) and STAT1^-/- ^splenocytes (lanes 5 and 15), arguing against an association between MMTV LTR translocation and mammary tumorigenesis in STAT1^-/- ^mammary glands. Click here for file

Additional file 2 **Supplementary Figure **2. **Establishment of Spontaneous STAT1^-/- ^Mammary (SSM) epithelial tumor cell lines**. **(A) **Primary STAT1^-/- ^mammary tumors were mechanically dissociated and then digested in collagenese solution. Disaggregated tumor and stromal cells were analyzed for the expression of cytokeratin as a marker for epithelial cells (green) and vimentin as a marker for mesenchymal cells (red) by immunofluoresence. Freshly disaggregated tumors were comprised of epithelial tumor cells (green) and stromal fibroblasts (red). **(B, C, and D) **Epithelial tumor cell lines SSM1 **(B)**, SSM2 **(C)**, and SSM3 **(D) **are devoid of stromal fibroblasts as evidenced by the complete absence of vimentin-positive cells. Representative images from 8 independent experiments. Click here for file

Additional file 3 **This file contains the expression values of 96 genes that were used in Figure **8**to classify 232 human breast cancer datasets, 13 mouse mammary models datasets and primary STAT1^-/- ^mammary tumors**. Click here for file
