# Supplementary figures and images for: STAT1-deficient mice spontaneously develop estrogen receptor α-positive luminal mammary carcinomas
Source: Breast Cancer Res. 2012 Jan 20;14(1):R16. doi: 10.1186/bcr3100 (PMC3496133; doi:10.1186/bcr3100)

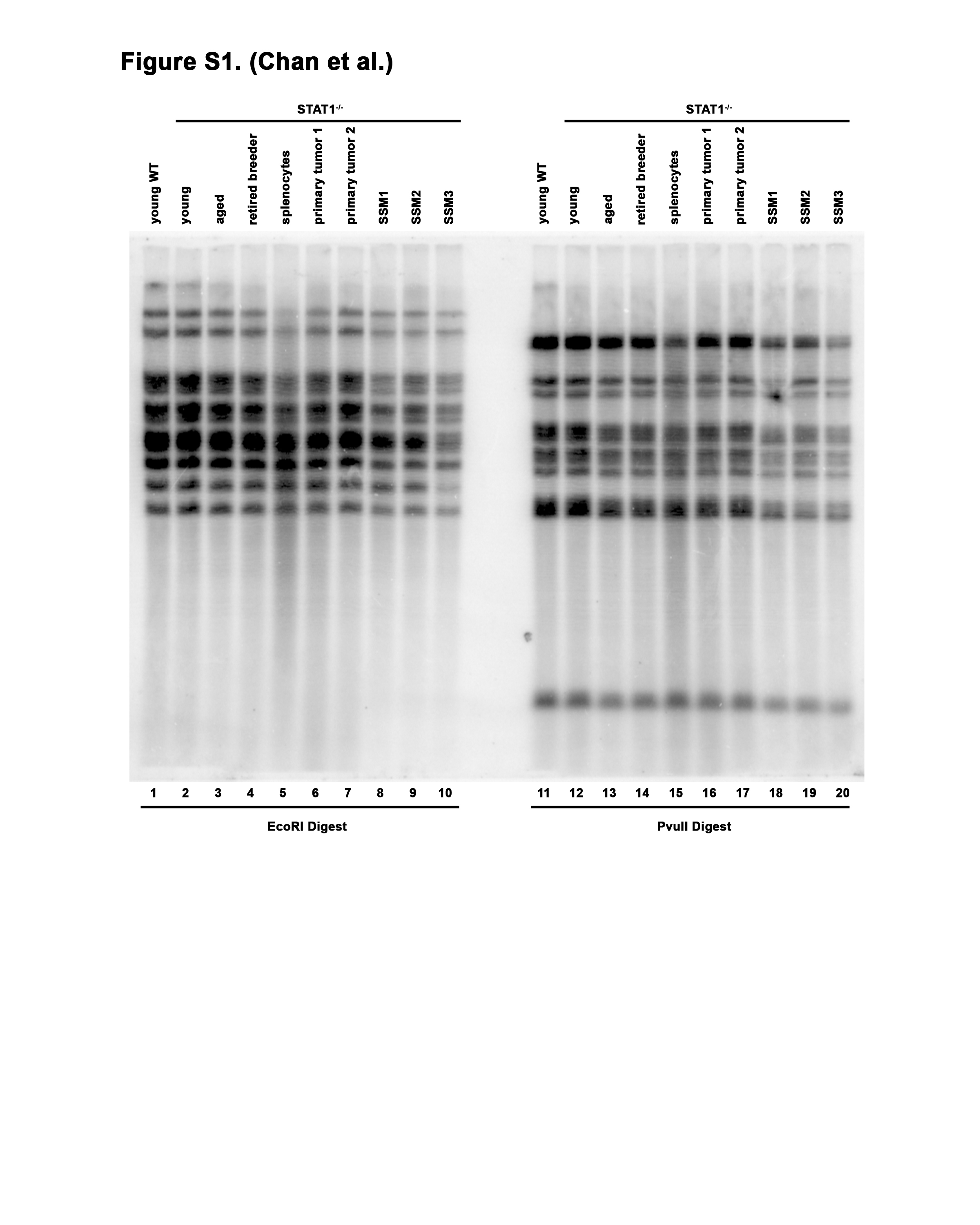

Supplement: Additional file 1 — Supplementary Figure 1. Evidence against translocation of endogenous murine mammary tumor virus (MMTV) as the cause of STAT1-/- mammary tumorigenesis. Southern blot analysis was used to detect the translocation of MMTV long terminal repeats (LTR). Genomic DNA was harvested from nontransformed mammary glands of young or aged nulliparous STAT1-/- mice (lanes 2, 3, 12, and 13), nontransformed mammary glands of retired STAT1-/- breeders (lanes 4 and 14), primary STAT1-/- mammary tumors (lanes 6, 7, 16, and 17), or SSM cell lines established from primary STAT1-/- mammary tumors (lanes 8 to 10 and lanes 18 to 20) and digested with either EcoRI (lanes 1 to 10) or PvuII (lanes 11 to 20). The number and the sizes of the DNA fragments hybridized to the MMTV LTR in these samples are indistinguishable from those in WT mammary glands (lanes 1 and 11) and STAT1-/- splenocytes (lanes 5 and 15), arguing against an association between MMTV LTR translocation and mammary tumorigenesis in STAT1-/- mammary glands. [file bcr3100-S1.JPEG]

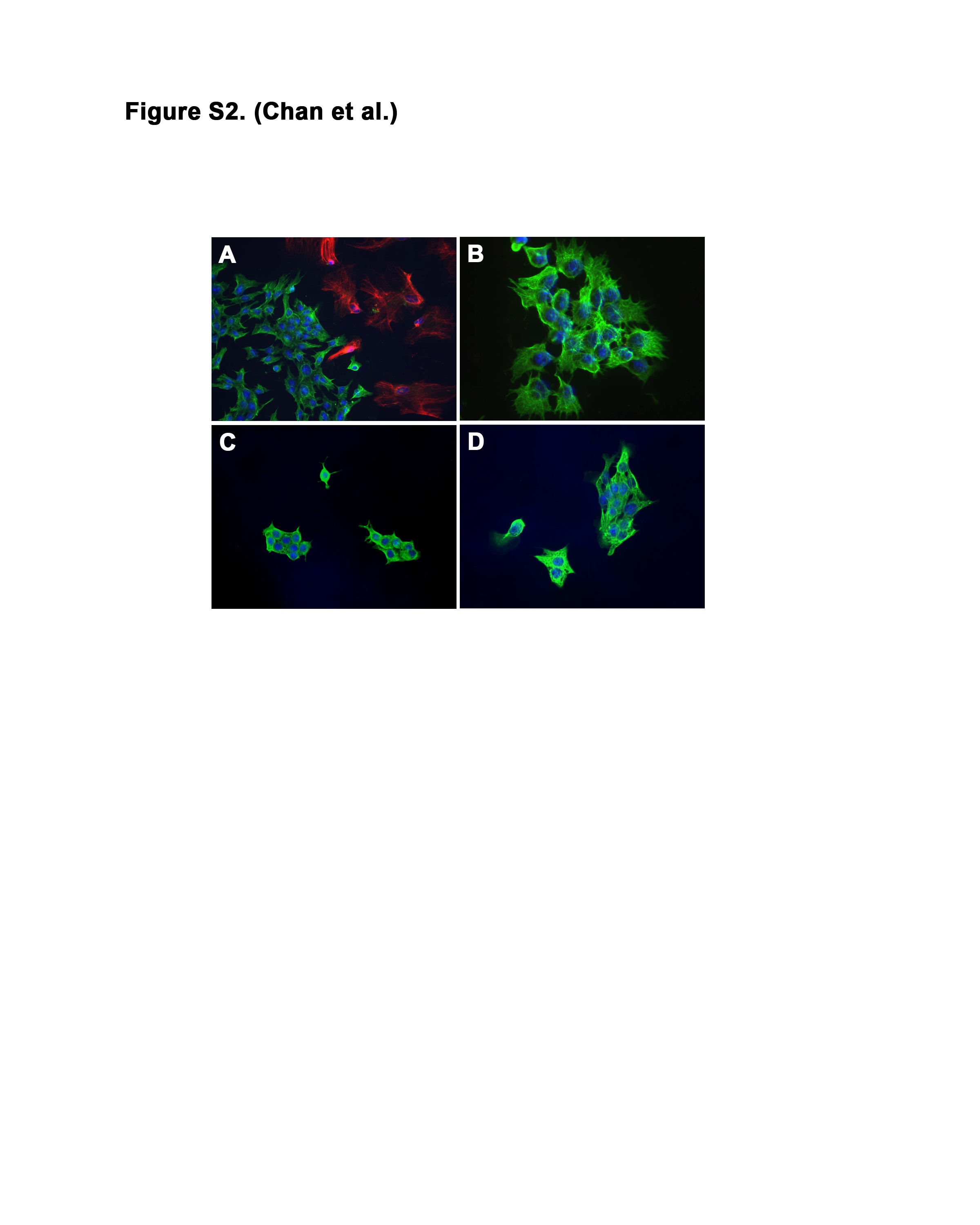

Supplement: Additional file 2 — Supplementary Figure 2. Establishment of Spontaneous STAT1-/- Mammary (SSM) epithelial tumor cell lines. (A) Primary STAT1-/- mammary tumors were mechanically dissociated and then digested in collagenese solution. Disaggregated tumor and stromal cells were analyzed for the expression of cytokeratin as a marker for epithelial cells (green) and vimentin as a marker for mesenchymal cells (red) by immunofluoresence. Freshly disaggregated tumors were comprised of epithelial tumor cells (green) and stromal fibroblasts (red). (B, C, and D) Epithelial tumor cell lines SSM1 (B), SSM2 (C), and SSM3 (D) are devoid of stromal fibroblasts as evidenced by the complete absence of vimentin-positive cells. Representative images from 8 independent experiments. [file bcr3100-S2.JPEG]
